# Frequency of Diarrhea, Stool Specimen Collection and Testing, and Detection of *Clostridioides Difficile* Infection Among Hospitalized Adults in the Muenster/Coesfeld Area, Germany

**DOI:** 10.1007/s00284-022-03143-6

**Published:** 2022-12-16

**Authors:** Natalie Effelsberg, Meike Buchholz, Stefanie Kampmeier, Andrea Lücke, Vera Schwierzeck, Frederick J. Angulo, Gordon Brestrich, Catherine Martin, Jennifer C. Moïsi, Christof von Eiff, Alexander Mellmann, Lutz von Müller

**Affiliations:** 1 Institute of Hygiene, University Hospital Münster, Robert-Koch-Str. 41, 48149 Münster, Germany; 2 Institute of Laboratory Medicine, Microbiology and Hygiene, Christophorus Kliniken, Südring 41, 48653 Coesfeld, Germany; 3 Medical Development and Scientific/Clinical Affairs, Pfizer Vaccines, 500 Arcola Road, Collegeville, PA 19426 USA; 4 Pfizer Pharma GmbH, Linkstr. 10, 10785 Berlin, Germany; 5 National Reference Center for C. Difficile, Münster, Germany

## Abstract

*Clostridioides difficile* infection (CDI) often manifests as diarrhea, particularly in adults of older age or with underlying comorbidities. However, only severe cases are notifiable in Germany. Moreover, failure to collect a stool specimen from inpatients with diarrhea or incomplete testing may lead to underdiagnosis and underreporting of CDI. We assessed the frequency of diarrhea, stool specimen collection, and CDI testing to estimate CDI underdiagnosis and underreporting among hospitalized adults. In a ten-day point-prevalence study (2019–2021) of nine hospitals in a defined area (Muenster/Coesfeld, North Rhine-Westphalia, Germany), all diarrhea cases (≥ 3 loose stools in 24 h) among adult inpatients were captured via medical record screening and nurse interviews. Patient characteristics, symptom onset, putative origin, antibiotic consumption, and diagnostic stool sampling were collected in a case report form (CRF). Diagnostic results were retrieved from the respective hospital laboratories. Among 6998 patients screened, 476 (7%) diarrhea patients were identified, yielding a hospital-based incidence of 201 cases per 10,000 patient-days. Of the diarrheal patients, 186 (39%) had a stool sample collected, of which 160 (86%) were tested for CDI, meaning that the overall CDI testing rate among diarrhea patients was 34%. Toxigenic *C. difficile* was detected in 18 (11%) of the tested samples. The frequency of stool specimen collection and CDI testing among hospitalized diarrhea patients was suboptimal. Thus, CDI incidence in Germany is likely underestimated. To assess the complete burden of CDI in German hospitals, further investigations are needed.

**Supplementary Information:**

The online version contains supplementary material available at 10.1007/s00284-022-03143-6.

## Introduction

*Clostridioides difficile* is an anaerobic, Gram-positive, spore-forming bacterium that can often be found in the human intestinal tract. It is acquired via the fecal–oral route and frequently colonizes the human gut [1]. However, when the microbial composition in the gut is disrupted, toxin-producing strains can lead to *C.* *difficile* infection (CDI). Although the main clinical manifestation of CDI is mild to severe diarrhea, CDI can result in a broad disease spectrum including severe outcomes such as pseudomembranous colitis, toxic megacolon, and death [2]. The 30 day mortality rate of CDI is up to 13% [3]. Furthermore, CDI has a high probability for recurrent infections [4]. The median global recurrence rate is 17% but differs remarkably between geographical regions and rates of up to 64% have been reported [5]. The major risk factor for CDI is use of antibiotics, especially clindamycin, cephalosporins, fluoroquinolones [6], and carbapenems [7]. Other risk factors include advanced age, hospitalization or living in a care facility, certain drugs (e.g. proton-pump inhibitors), feeding tubes, being female, and several comorbidities [5, 8]. Although CDI is the leading cause of healthcare-associated diarrhea, reports about community-associated CDI cases are increasing as well [9].

In Germany, only severe CDI cases are nationally notifiable to public health officials in accordance with the German Infection Protection Act (IfSG). A severe case is defined as (i) outpatient infection requiring hospitalization, (ii) intensive care unit treatment, (iii) surgical intervention, or (iv) death with CDI [10]. Among the 16 federal states in Germany, Saxony is the only state with mandatory reporting of infectious gastroenteritis and its causative agents including *C. difficile* [11]. Germany does mandate, however, that every hospital conducts surveillance for nosocomial infections. Furthermore, many hospitals voluntarily participate in the national surveillance system for nosocomial infections, called hospital-infection-surveillance-system (KISS), which is operated by the national reference center for surveillance of nosocomial infections (NRZ). KISS includes a module for *Clostridium-difficile*-associated diarrhea (CDAD-KISS) [12]. According to this reporting system, the hospital-based CDI incidence in participating hospitals increased from 6.1 CDI cases per 10,000 patient days in 2007 to 7.4 in 2015 and then declined to 4.5 CDI cases per 10,000 patient days in 2020 [13]. Estimates of hospital-based CDI incidence can also be obtained from the health insurance accounting data using hospital diagnoses (ICD-10 codes) including *C. difficile* enterocolitis (ICD-10 A04.7) [14].

According to guidelines of the German Commission for Hospital Hygiene and Infection Prevention (KRINKO), all patients with hospital-onset diarrhea of unknown origin should have a stool specimen collected and tested for CDI [15]. These guidelines are similar to other national and international recommendations such as the s2k-guideline for gastrointestinal infections [16], the CDI diagnostic guidelines by the European Society of Clinical Microbiology and Infectious Diseases (ESCMID) [17], and guidelines from the Infectious Diseases Society of America (IDSA) [18]. Despite these recommendations, the decision to collect and test a stool specimen is at the discretion of the attending physician.

To evaluate the potential undertesting and underreporting of CDI in hospitals in Germany, we conducted a multi-center point-prevalence study assessing the prevalence of diarrhea, the proportion of stool specimens collected and tested for CDI, and CDI rates among hospitalized adults.

## Materials and Methods

A point-prevalence study was performed at nine of the ten hospitals in the Muenster/Coesfeld area in North-Rhine Westphalia, Western Germany. The study area comprised an urban area, the city of Münster, and a more rural area, the county of Coesfeld, and had a population of 537,115 in 2020 [19]. The participating hospitals included 95% (3431/3612) of hospital beds in these areas. All patients in all adult wards in each participating hospital were observed for ten consecutive working days sequentially between October 2019 and June 2021. Seven hospitals were visited prior to the SARS-CoV-2 pandemic and two during the pandemic in periods with low local COVID-19 incidence (September 2020 and June 2021).

During the observation period, all hospitalized adults were prospectively screened for diarrhea, which was defined as ≥ 3 loose stools (Bristol stool types 5–7) within 24 h. Patients with diarrhea onset prior to hospitalization were included. However, every patient was only recorded once per hospital stay, even if diarrhea symptoms reoccurred. To identify inpatients with diarrhea, medical records were screened and nurses on every ward were interviewed daily. A case report form (CRF) was completed for each adult inpatient with diarrhea, including questions on patient demographics, onset of diarrhea, and current antibiotic intake. After case identification, diarrhea progress was tracked in the patient’s CRF. The CRF also recorded whether a standard-of-care stool sample was collected or planned to be collected. Furthermore, hospital staff were asked to state the putative cause of diarrhea and to provide a reason if a stool sample was not collected or planned. In addition, laboratory results of all diagnostic stool tests conducted in the observation periods (+one week) were obtained and results were matched with the CRFs using the case IDs as identifiers. All laboratories tested for CDI in a two-step approach. First, glutamate dehydrogenase (GDH) and toxin A/B testing was performed using either an enzyme immunoassay (EIA) (C. Diff Quik Check Complete, Alere, Köln, Germany) or chemiluminescent immunoassay (CLIA) (DiaSorin, Saluggia, Italy). Discrepant results were resolved in a nucleic acid amplification technique (NAAT) test targeting the toxin-encoding genes. Samples that were negative by NAAT were classified as CDI negative.

Using the time difference between diarrhea onset and hospitalization, diarrhea was classified as either community-onset (diarrhea onset before or within the first day after hospitalization) or hospital-onset (diarrhea onset ≥ 2 days after hospitalization).

## Results

### Study Population and Patient Characteristics

The number of beds in each of the nine observed hospitals ranged from 92 to 1209 (median: 273) beds. In total, 6998 hospitalized adults and 23,705 patient-days (average length of stay = 3.4 days) were reported during the observation period. Of the hospitalized adults, 3689 (53%) were women, 3308 (47%) men, and one non-binary. Among all adults, 1840 (26%) were aged younger than 50 years.

**Fig. 1:**
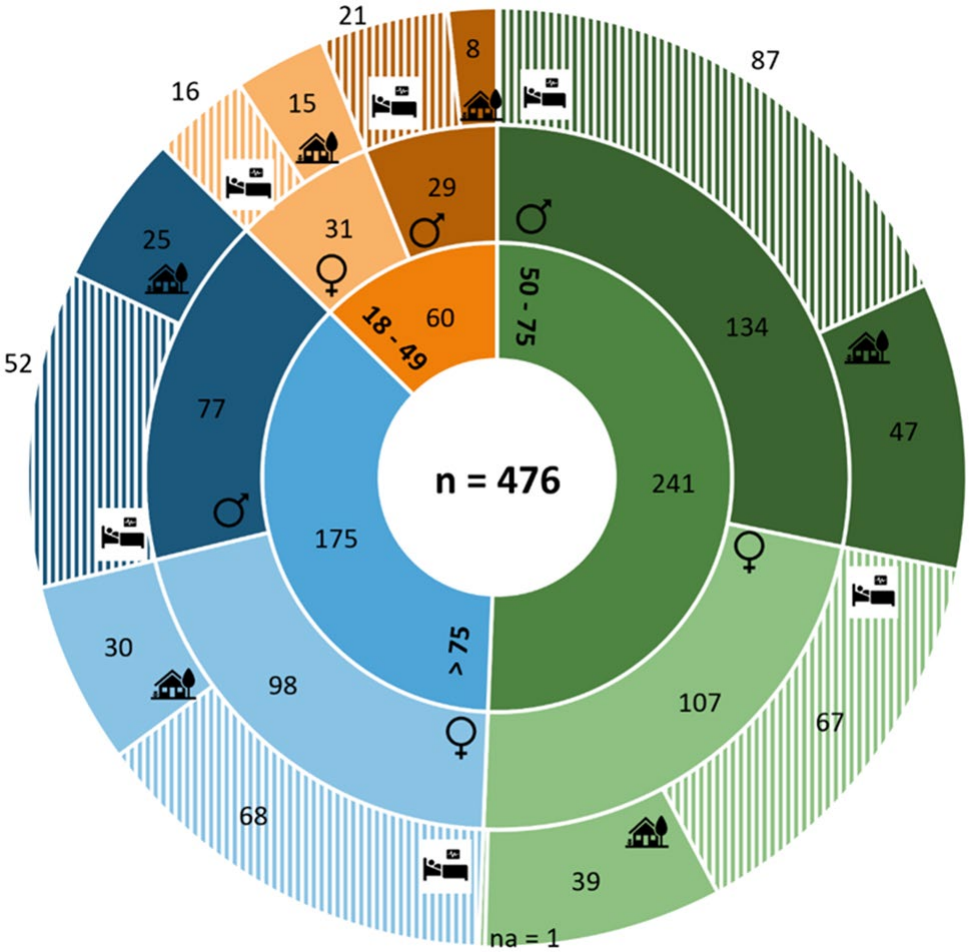
Number of patients with diarrhea stratified by age group, gender, and diarrhea onset location. Inner ring = Age group, middle ring = gender (darker shade = male, lighter shade = female), Outer ring = epidemiological classification (solid = community onset, striped = hospital onset) (Color figure online)

During the observation periods, 476 patients with diarrhea (≥ 3 loose stools in 24 h) were identified, which is equivalent to 7% of all admissions and an incidence of 201 diarrhea cases per 10,000 patient days. Of the 476 diarrhea patients, 416 (87%) were aged 50 years and older with a median age of 69 years (IQR: 57–80 years). Among these 476 patients, 240 (50.4%) were men and 236 (49.6%) were women. Of the 476 inpatients with diarrhea, 164 (34%) were classified as community-onset and 311 (65%) as hospital-onset diarrhea, while the date of onset was missing for one patient. Figure 1 shows the 476 diarrhea patients stratified by gender and age group.

**Fig. 2:**
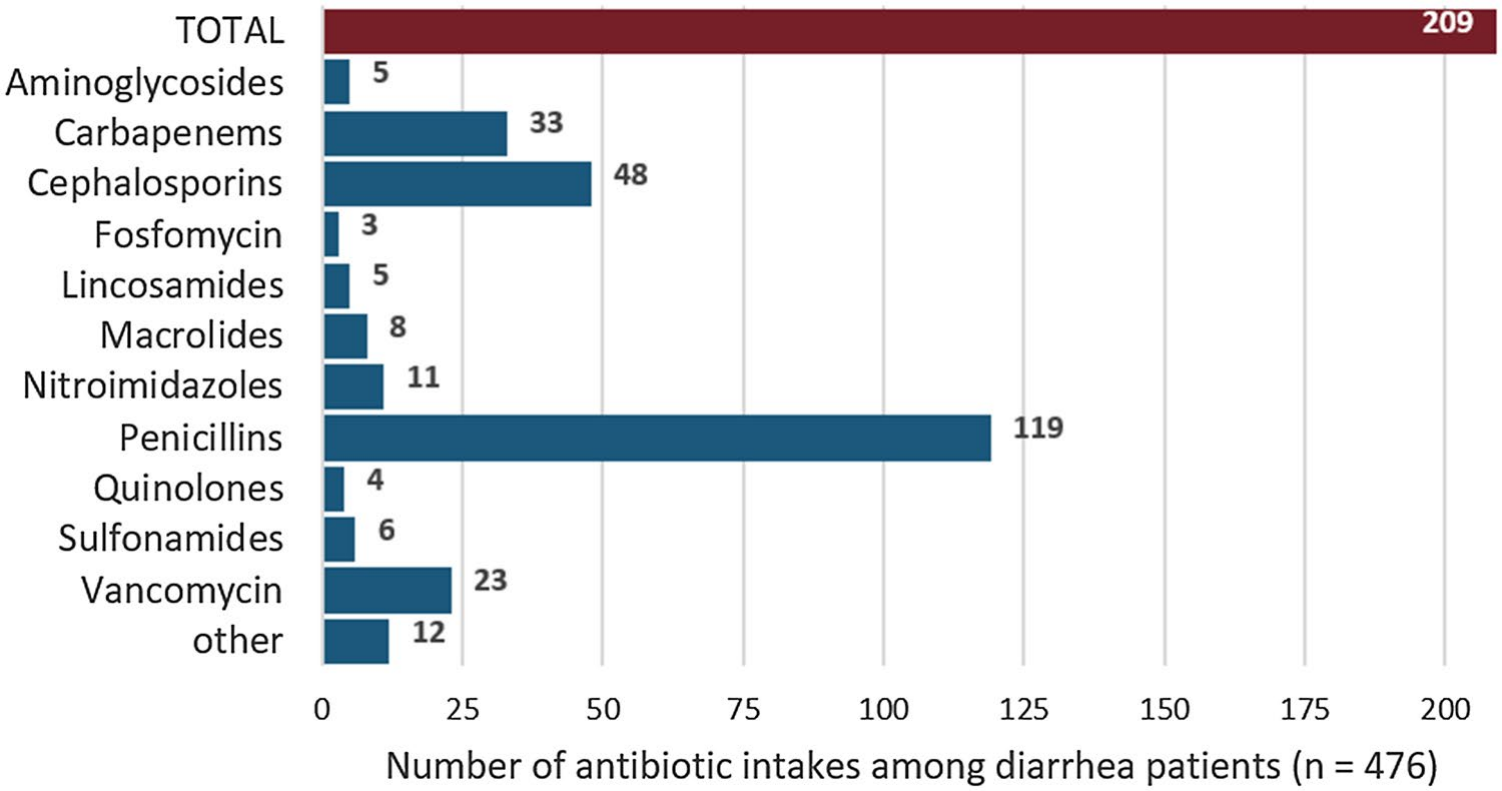
Number of individual prescriptions by classes of antimicrobial agents used for the treatment of 476 patients with diarrhea. Of the 209 diarrhea patients treated with antibiotics during the observation period, 52 patients were prescribed multiple (2–5) antibiotics from different classes

Among the 476 diarrhea patients, 209 (44%) were treated with antibiotics within the observation period; 52 (29%) of these received more than one antimicrobial agent (2–5, median = 2). An overview of the antimicrobial classes used is shown in Fig. 2.

### Frequency of Stool Sample Collection

**Fig. 3:**
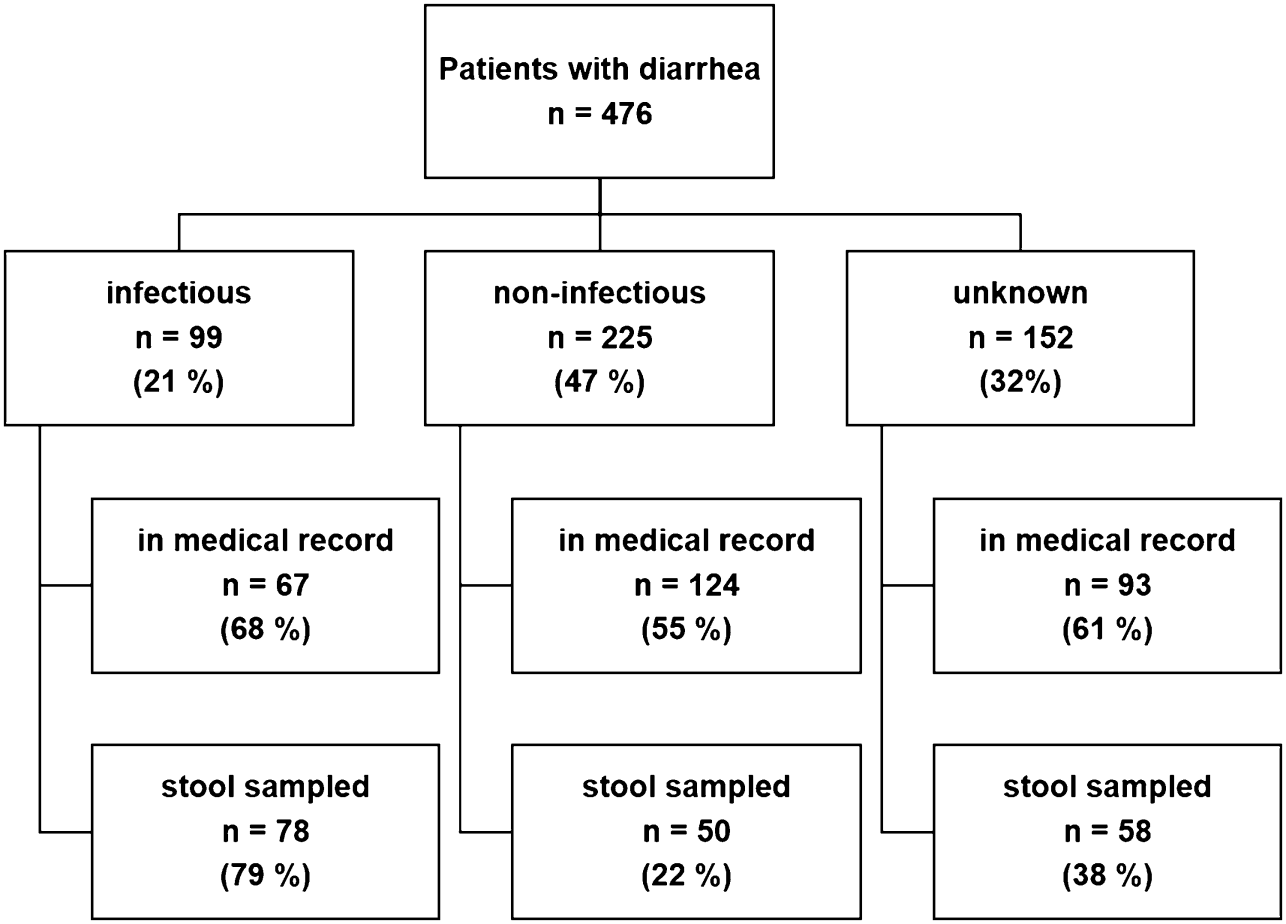
Number of diarrhea cases that were documented in the medical records and number of stool samples taken stratified by presumable diarrhea origin

**Fig. 4:**
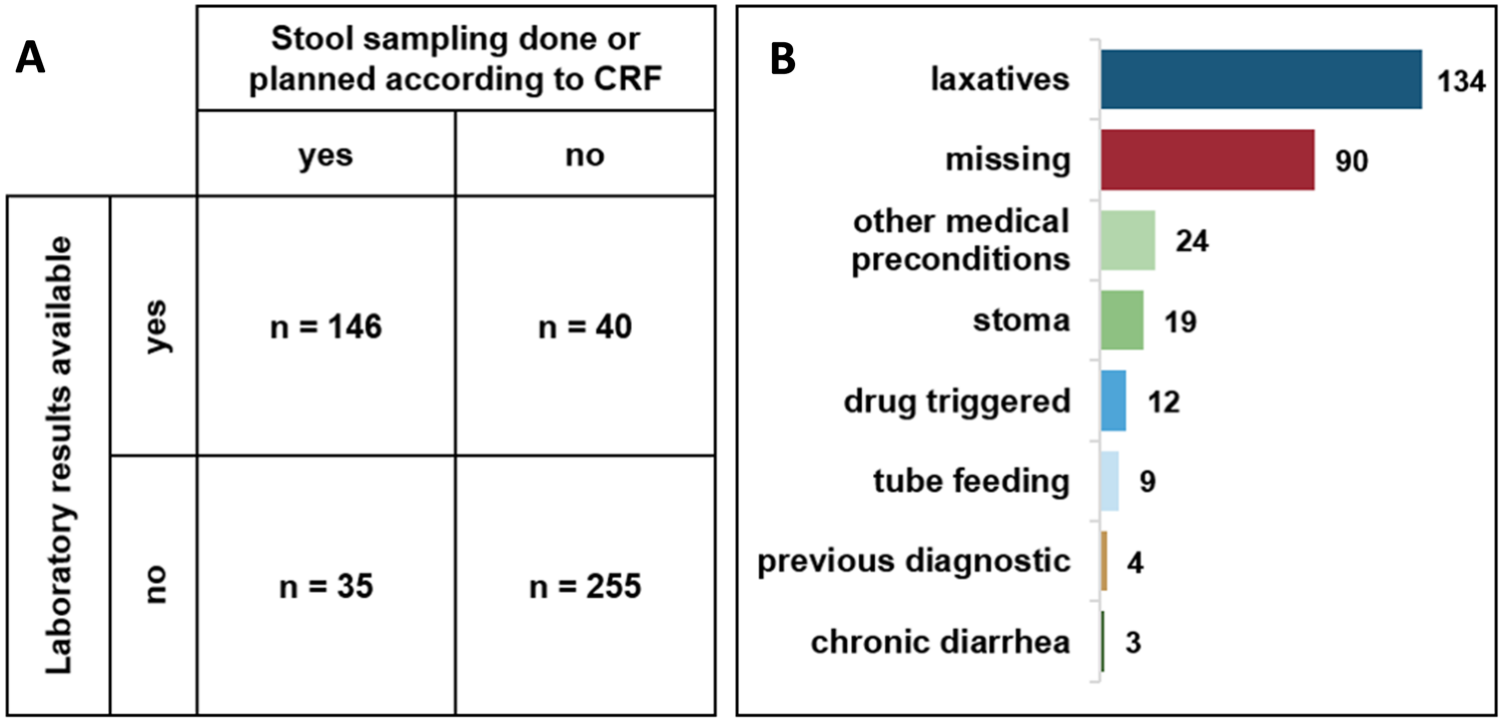
Number of cases where stool samples were taken or planned according to the CRF and number of cases where samples were actually tested in the laboratory (**A**). For the 295 cases where no samples were taken or initially planned, the reason stated in the CRF is listed (**B**)

Of the 476 diarrhea patients, 192 (40%) were only identified via active nurse interviews, i.e. diarrhea was not entered in the medical record. The documentation and stool sampling rate differed between cases with presumably infectious, non-infectious, or unknown diarrhea origin (Fig. 3). According to the study CRF, no stool specimen was collected (or planned to be collected) from 62% (295/476) of inpatients with diarrhea. However, there were several discrepancies between the CRF and the number of matching results in the laboratory reports (Fig. 4a). The reasons for not taking (or planning to take) a stool sample are presented in Fig. 4b.

Of the 186 samples, for which laboratory stool diagnostics were performed, 40 (22%) were tested only for CDI, 26 (14%) only for other pathogens, and 120 (65%) for both; therefore, only 34% (160/476) of inpatients with diarrhea were tested for CDI.

### Hospital-Based CDI Incidence

CDI was detected in 18 (11%) of the tested patients, while 10 (7%) samples were positive for other pathogens (Enteropathogenic *Escherichia coli*: *n* = 3, *Campylobacter*: *n* = 2, *Salmonella*: *n* = 1, Norovirus: *n* = 4). The hospital-based CDI incidence was 7.6 CDI cases per 10,000 patient-days. Of the 18 CDI cases detected, 17 were found in patients older than 50 years (median: 70 years). CDI was diagnosed in 3 (17%) patients with community-onset diarrhea and 15 (83%) patients with hospital-onset diarrhea, and 14 (78%) CDI cases correlated with antibiotic intake. With regards to putative diarrhea causes as stated by hospital staff, CDI was confirmed in 11 (61%) samples with putative infectious, 2 (11%) with putative non-infectious, and 5 (28%) with unknown diarrhea origin.

## Discussion

In this hospital-based cross-sectional study, we showed that diarrhea is common among hospitalized adults, especially those 50 years of age or older, and affects 7% of patients at a rate of 2% per patient-day of hospitalization. Within the nine participating hospitals, we found a hospital-based CDI incidence of 7.6 cases per 10,000 patient days. The voluntary surveillance system CDAD-KISS receives data from 27% of German hospitals. In 2019 and 2020, 27,615 and 22,426 CDI cases were reported, respectively, which corresponds to an incidence of 4.5 – 4.8 cases per 10,000 patient days in the CDAD-KISS participating hospitals [13]. Thus, we found a higher hospital-based CDI incidence in our study than that reported in CDAD-KISS in the years where the study was conducted. A possible explanation for this deviation might be under-reporting due to under-recognition of symptomatic diarrhea in CDAD-KISS. In our study, a substantial number of patients with diarrhea was only identified by active nurse interviews, which would have been missed by routine hospital surveillance relying on medical records only. It is also possible that the focused assessment of diarrhea over a short period of time may have introduced a bias by increasing awareness among hospital staff and thus increased the sampling and consequently the CDI detection rate. Another reason might be regional biases. As the national coverage of CDI surveillance is relatively low, only hospital-based but not population-based incidences were inferred, which might differ between various regions.

Estimates of the hospital-based CDI incidence have also been assessed in different studies in Germany. In a prospective study conducted in a 1200-bed tertiary care university teaching hospital in Saarland, a federal state south of our study area, in 2013, where all diarrhea patients were actively tested for CDI, the hospital-based CDI incidence was 12.5 cases per 10,000 patient-days [20]. In a similar study conducted in 40 hospitals in Hesse, a federal state in central Germany, between 2011 and 2013, the average hospital-based CDI incidence was 9.9 cases per 10,000 patient-days [21]. In 2012–2013, the 87 hospitals in Germany that participated in the multi-country EUCLID study reported a hospital-based CDI incidence of 21.7–27.9 per 10,000 patient-days [22]. The EUCLID study also estimated that the hospital-based CDI incidence may be under-estimated by 25%, primarily due to failure to test diarrheal stool specimens and inadequate laboratory testing.

In our study, we investigated under-sampling as a potential source for under-diagnosis of CDI. Only 39% of all identified diarrhea patients had a stool sample sent to the lab and just 34% were tested for CDI. When asked for reasons for the absence of laboratory diagnostics, a presumably non-infectious cause was reported for the majority of cases with laxatives being the most reported reason. However, for one third of the patients, no rationale was provided. Among these cases with missing reasons, 78% were hospital-onset diarrhea cases that could have resulted from nosocomial infection (Fig. 1). According to European and national guidelines, it is recommended that any hospitalized adult with diarrhea should be tested for CDI if no other cause is known [15]. However, this can be problematic as diarrhea may be multicausal, with for example both laxative use and an infectious etiology contributing to clinical symptoms. Further, certain non-infectious causes of diarrhea such as inflammatory bowel disease are known risk factors for the acquisition of CDI [23–25]. In our study, 11% of CDI cases were detected in patients with presumed non-infectious diarrhea and 28% in patients with unknown diarrhea origin. These numbers suggest that there might be more missed cases of CDI in patients that were not sampled. Thus, we emphasize the need to assess the presence of CDI among patients with presumably non-infectious diarrhea. The high number of unsampled diarrhea cases with unknown origin (62%) also indicates that current guidelines are not well implemented.

Our study also found poor documentation of episodes of diarrhea in the medical records, which may be related to a lack of specific guidelines on the need to document diarrhea cases. In addition to potential under-sampling, inadequate documentation can impede infection control measures such as contact precautions. This problem does not seem to be exclusive to German guidelines as the potential under-diagnosis of CDI due to poor sampling efforts has been reported similarly by the EUCLID study for other European countries [22, 26]. In the US, it was shown that only 32% of new-onset diarrhea cases in hospitals and long-term facility care centers in Louisville, Kentucky had a stool specimen collected [27]. Though the real number of CDI among the diarrhea cases that were not sampled cannot be inferred from our data, given the low rate of sampling and poor documentation of putative causes, it is very likely that the CDI burden in Germany is higher than currently assumed. Moreover, we did not assess the potential impact of inadequate laboratory assays on CDI under-diagnosis. All laboratories in our study performed toxigenic *C. difficile* testing in a 2-step approach, where discrepant results were resolved by NAAT, as recommended by the ESCMID [17]. We would expect only minor discrepancies between laboratories using standardized assays according to current guidelines. However, other approaches may lead to under-diagnosis or over-diagnosis of CDI as shown by the EUCLID study [22]. Another factor that was not assessed in our study is the impact of community-treated cases on the overall CDI burden, which is currently unknown because CDI surveillance is focused on hospitalized cases.

In addition to CDI prevalence, background data were collected from diarrhea patients to assess possible risk factors. Our study confirms known risk factors for CDI acquisition as the majority of the 18 CDI cases we found were hospital-onset and associated with high age and antibiotic intake. In the CRF, only antibiotic substances but not the timing of intake were retrieved, which makes it difficult to differentiate between antibiotics as putative CDI causes and those used for CDI treatment. However, a relatively high number of antibiotics that are known to be associated with CDI, e.g. carbapenems and cephalosporins, were administered.

## Conclusion

In conclusion, we have identified a high prevalence of diarrhea among hospitalized adults. Despite national and international recommendations on CDI testing of nosocomial diarrhea, the frequency of stool specimen testing was low, raising the possibility of CDI under-diagnosis. Furthermore, the rationale for lack of testing was not well documented. This point-prevalence study is limited by the short observation periods. Although the observation windows were distributed over different seasons over the course of 1.5 years, longer periods would be needed to collect reliable population-based hospitalized CDI incidence data. Furthermore, only standard-of-care stool samples were investigated but for a full picture every diarrheal stool sample should be tested. Therefore, we suggest that further investigations including CDI testing for all new-onset diarrhea patients would provide precise estimates of the CDI incidence and helpful insights into the level of CDI under-diagnosis within hospitalized adults in Germany.

## Supplementary Information

Below is the link to the electronic supplementary material.

Supplementary file1 (XLSX 42 KB)

## Data Availability

The data that support the findings of this study are available in Supplemental table 1.
